# Genome Sequence of a Plant-Associated Bacterium, *Bacillus amyloliquefaciens* Strain UCMB5036

**DOI:** 10.1128/genomeA.00111-13

**Published:** 2013-03-21

**Authors:** Shahid Manzoor, Adnan Niazi, Sarosh Bejai, Johan Meijer, Erik Bongcam-Rudloff

**Affiliations:** a Department of Animal Breeding and Genetics, SLU Global Bioinformatics Centre, Swedish University of Agricultural Sciences, Uppsala, Sweden; b Department of Plant Biology and Forest Genetics, Uppsala Biocenter, Swedish University of Agricultural Sciences and Linnéan Center for Plant Biology, Uppsala, Sweden; c University of the Punjab, Lahore, Pakistan

## Abstract

We announce here the genome sequence of *Bacillus amyloliquefaciens* strain UCMB5036, a plant growth-promoting bacterium isolated from a cotton plant. Its genome contains gene clusters involved in nonribosomal synthesis of secondary metabolites known for their antimicrobial activities. The availability of this genome will provide novel insights into plant-bacterium–associated activities.

## GENOME ANNOUNCEMENT

*Bacillus amyloliquefaciens* strain UCMB5036 is a Gram-positive rhizobacterium that is an important tool for studies of plant-bacterium associations and its role in plant growth promotion through the production of stimulating compounds and the suppression of soilborne pathogens by synthesizing antimicrobial and antifungal metabolites or priming plant-induced systemic resistance. It was isolated from the inner tissues of the cotton plant (*Gossypium barbadense*) in Tajikstan and identified as a member of the *B. amyloliquefaciens* group based on phenotypic analysis (1). The strain has shown great potential for the promotion of plant growth and the prevention of diseases in oilseed rape (*Brassica napus*) (2) and *Arabidopsis thaliana* (unpublished data).

Genomic DNA was extracted using a QIAmp DNA mini kit (Qiagen). Short paired-end reads of 75 bp in length with an average insert size of 230 bp were generated from two sequencing lanes, comprising 6,544,152 and 6,819,744 reads, by using Illumina multiplexed sequencing technology. Whole-genome assembly of the *B. amyloliquefaciens* UCMB5036 genome was accomplished with a comparative genome assembly method (3), which combines *de novo* and mapping assemblies. The paired-end reads from lane 1 were fed into MIRA version 3.4 (4) for both mapping and *de novo* assembly, and read data from lane 2 were provided to Velvet version 1.1.04 *de novo* assembler (5). Mapping assembly was done against the available genome of *B. amyloliquefaciens* FZB42 (accession no. NC_009725) (6). Contigs produced through *de novo* assembly of read data from both lanes were sorted and oriented along the reference genome and then aligned to the mapping assembly using Mauve genome alignment software (7). Alignment of contigs to mapping assembly indels covered all the gaps in the genome. Those covered gaps were all verified through PCR amplification using a Hot Start high-fidelity DNA polymerase (Phusion, Thermo Scientific) and subsequent Sanger sequencing (Macrogen).

*B. amyloliquefaciens* UCMB5036 has a circular chromosome of 3,910,324 bp with 46.60% G+C content. Structural and functional annotations were accomplished via the Magnifying Genome (MaGe) annotation platform (8). In total, 3,660 coding sequences (CDSs) were predicted using embedded gene prediction tools. In total, 89 tRNA genes and 10 rRNA operons were identified using tRNAscan-SE version 1.23 (9) and RNAmmer version 1.2 (10), respectively. Putative functions of the encoding genes were assigned automatically by MaGe´s inbuilt BLASTp searches against the UniProt databank. The predictions were reviewed and curated manually to avoid false forecasts.

The *B. amyloliquefaciens* UCMB5036 genome contains 3,842 predicted open reading frames (ORFs), of which 95.39% of the genetic proportion had counterparts in the FZB42 genome; this suggests a high degree of gene synteny to the reference genome. The genomic sequence of *B. amyloliquefaciens* UCMB5036 confirmed the presence of nonribosomal peptide synthetase (NRPS) and polyketide synthase (PKS) gene clusters: surfactin (*srf*), fengycin (*fen*), difficidin (*dfn*), bacilysin (*bac*), macrolactin (*mln*), bacillaene (*bae*), bacillomycin D (*bmy*), and bacillibactin (*dhb*). These are responsible for the synthesis of secondary metabolites, including antifungal and antibacterial compounds (6). The genome also contains genes involved in the catabolism of plant-derived compounds, resistance to heavy metals and drugs, motility and chemotaxis, root colonization, and other functions that presumably give the bacterium an advantage in developing a symbiotic relationship with plants.

### Nucleotide sequence accession number. 

The complete nucleotide genome sequence of *B. amyloliquefaciens* UCMB5036 has been deposited in the European Nucleotide Archive (ENA) database under the accession no. HF563562.
